# Where are higher-order cognitive functions? The paradox of non-locality in awake cognitive mapping using a complex dynamic system framework

**DOI:** 10.3389/fpsyg.2025.1542505

**Published:** 2025-03-03

**Authors:** Jesús Martín-Fernández, Nayra Caballero-Estebaranz, Esteban Félez, Natalia Navarro-Peris, Pedro Pérez del Rosario, Raúl Hernández Bisshopp, Jaime Domínguez-Báez

**Affiliations:** ^1^Department of Neurosurgery, Nuestra Señora de Candelaria University Hospital, Tenerife, Spain; ^2^Department of Cognitive-Affective Neuroscience, e-Awake Institute, Tenerife, Spain; ^3^Canary Association of Creative Therapies (ASCATEC), Tenerife, Spain; ^4^Faculty of Biomedical and Health Sciences, Universidad Europea de Canarias, Tenerife, Spain; ^5^Institute of Neuroinformatics: University of Zürich and ETH, Zürich, Switzerland

**Keywords:** cognitive mapping, computational neurosciences, awake neurosurgery, emotions, network neuroscience, connectomics

## Abstract

This study addresses the challenge in identifying and preserving higher-order cognitive functions within a complex dynamic systems framework during neurosurgery. Traditionally, neurosurgical practice has prioritized avoiding language and motor deficits, while higher-order functions—such as social cognition and executive processes—remain underexplored. These functions arise from dynamic large-scale networks operating in an optimal balance between synchronization and metastability rather than from isolated and localized cortical regions. This complexity highlights a paradox of non-locality in awake cognitive mapping: no single area “contains” a function, but certain “critical points” can transiently disrupt network dynamics when stimulated intraoperatively. Direct electrical stimulation provides unique real-time insights by inducing brief dyssynchronizations that elicit observable behavioral changes, allowing neurosurgeons and neuropsychologists to pinpoint crucial cortical and subcortical “connectome-stop points” and minimize damage. Preserving deep white-matter tracts is essential, given their limited neuroplasticity and the profound, often irreversible impact of tract lesions on cognition. To address these challenges, we propose a three-step awake cognitive mapping approach: (1) localizing critical points of networks via DES-driven behavioral impairment, (2) constant monitoring of multiple cognitive domains as tumor resection progresses, and (3) halting resection at connectome-stop points to prevent irreversible deficits. An illustrative case involving a right parietal glioma demonstrates how this methodology integrates computational neuroscience, network theory, and clinical practice to achieve optimal functional preservation and maintain the patient’s quality of life.

*“Coordination in the brain is like a Balanchine ballet. Neural groups briefly couple, some join as others leave, new groups form and dissolve, creating fleeting dynamical coordination patterns of mind that are always meaningful but do not stick around for very long.”*—Kelso and Engstrom (Kelso and Engstrøm, 2006).

## Introduction

Where are higher-order functions located within the brain? Can we identify and preserve the critical regions responsible for cognitive-emotional functions *in vivo*? Why has neurosurgery mainly focused on avoiding language and motor deficits while we still observe up to 30–35% of long-lasting deficits in social cognition and other higher-order functions? (Duffau, 2021; Pertz et al., 2022).

One of the main reasons has been the prevalence of a rigid and modular vision (localizationism) of the central nervous system, tethering our understanding of the human brain to structure without recognizing that structure only influences but does not define function (Shine et al., 2016; Cabral et al., 2017). Shortly after Paul Broca described the case of Victor Louis Leborgne (Broca, 1861), other eminent neuroscientists began exploring ideas beyond a network perspective. For instance, Pierre Marie stated, “Patients could be aphasic not due to a lesion in a specific region of the brain, but because of a set of complex anatomical structures” (Brais, 1992). However, these papers did not have enough impact until network neuroscience emerged much later (Mandonnet and Duffau, 2021). Another significant reason is the persistent lack of understanding of the neural underpinnings of conation, cognition, and emotion—those functions that enable us to generate an immense repertoire of goal-directed behaviors in response to internal and external stimuli from an ever-changing world (Duffau, 2017; Martín-Fernández et al., 2024). In this domain, awake mapping and cognitive neuropsychology have been working to fill this gap, a challenge for neuropsychologists and neurosurgeons in the operating theater: Where and how should cognitive functions be located and preserved during surgery? Unlike some functions that are modular and based on local-level networks, such as top-down (motor functions) and bottom-up (visual and sensory), higher-order cognitive functions are context-sensitive functions that arise as emergent properties resulting from the interaction and reconfiguration of several dynamic large-scale networks (Shine et al., 2016; Herbet and Duffau, 2020). This complicates the identification of complex cognitive areas involved in these functions. Although we have attempted to rely on recent advances in neuroimaging, it is important to highlight its spatiotemporal limitations since fMRI processing depends on hemodynamic responses (the BOLD signal) and does not directly detect electrical activity or rapid metabolic changes across widespread brain regions (Ogawa et al., 1993; Menon et al., 1999).

Over the last decade, computational neuroscience has led us to a more integrative, systems-level understanding of the connectome provided by complex dynamic systems theory. Within this framework, the brain operates like a flock of starlings during a murmuration, where each brain node—part of several large-scale networks—interacts in a state of self-organized criticality, that is, between order and chaos (Shine et al., 2021; Cabral et al., 2022). This state is known in computational neuroscience as the optimal state of synchronization and metastability of the central nervous system (Váša et al., 2015). Altered metastability has been implicated in various psychiatric illnesses, such as Alzheimer’s disease or schizophrenia (Lee et al., 2018; Anyaeji et al., 2021). Though limited, these models can help us relate structural connectivity, neural dynamics, and behavior.

But how could neurosurgery go one step further? Here, we propose a perspective about how we could move to the emergentism of higher-order cognitive functions under the complex dynamic systems framework to better understand how to preserve those neural networks involved in conation, cognition, and emotion during awake mapping. For this purpose, three points will be the backbone of this perspective: (1) The brain understood from complex dynamic system theory; (2) the importance of white-matter tracts considered as stop points during the surgery; and (3) awake cognitive mapping in three steps showing an illustrative case.

## The paradox of non-locality: the uncertainty of critical regions of higher-order networks

Since Scott Sherrington’s experiments, which inspired Wilder Penfield in the 1950s (Feindel, 2007), it has been understood that the application of low-frequency input to the cerebral cortex can help identify areas responsible for modular functions such as movement. Therefore, neurosurgeons have historically associated DES with (1) transient motor or language impairment within a (2) discrete cortical area of less than 1 cm (Ojemann et al., 1989). This has led us to associate specific points within the brain with specific functions—a concept known as “one-to-one single mapping.” However, this direct association applies only to modular functions, such as sensorimotor functions, which depend on unimodal and localized networks (Herbet and Duffau, 2020). Since these are top-down or bottom-up functions, DES would generate disruption and consequent first-level neural disruption according to the three-level model recently proposed by Duffau (2020). This is not the case with higher-order cognitive functions, which are strongly interrelated (Königs et al., 2021) and provide *Homo sapiens* the flexibility to exhibit a plethora of goal-directed behaviors (Cabral et al., 2022; Vohryzek et al., 2022). These higher-order functions rely on large-scale neural networks that are partially delocalized and constantly reconfiguring.

**Figure 1:**
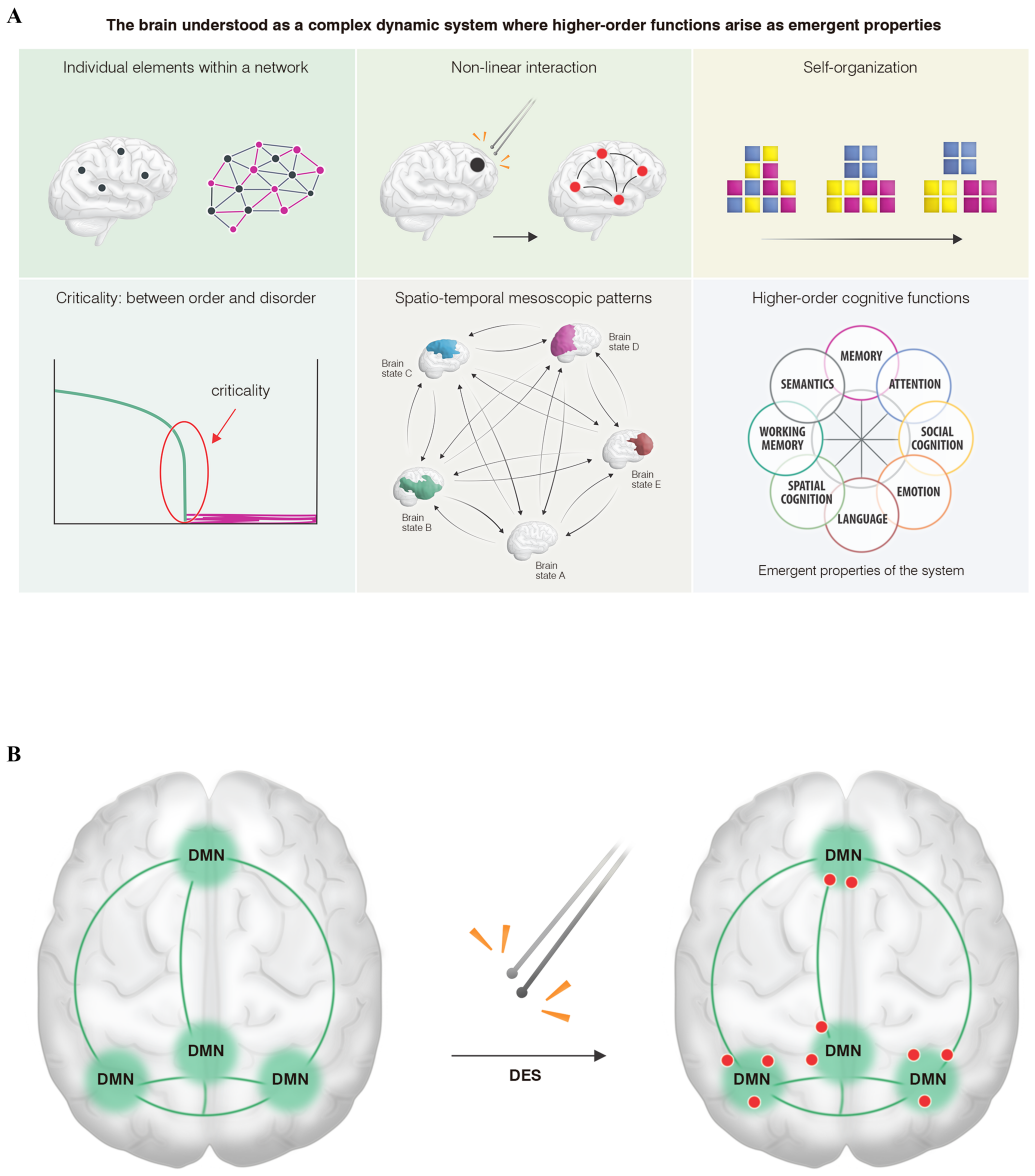
**(A)** From the perspective of complex dynamic systems theory, the human connectome, like other complex biological systems in nature, is characterized by several key features. (A) It is composed of individual elements that do not operate in isolation, but rather as networks governed by (B) non-linear interactions, tending (C) toward self-organization across different scales (micro-, meso-, and macroscopic). For optimal functioning, the system must remain (D) in a state of criticality (balanced between order and chaos), thus generating (E) mesoscopic patterns of connectivity—commonly referred to as “brain states”—that dynamically emerge across space and time within a metastable regime. (F) As a result, higher-order cognitive functions would arise as emergent properties of the system. **(B)** An illustrative model shows the main hubs of a higher-order cognitive network, specifically the default mode network: the ventromedial prefrontal cortex, temporoparietal junctions, and the precuneus-posterior cingulate cortex. When (DES) is applied within these hubs, only certain points (red dots) exhibit transient network distortions induced by low-frequency stimulation, resulting in behavioral impairments such as difficulties with theory of mind or emotion recognition. Rather than interpreting these discrete points as “containing” the cognitive function, they should be viewed as critical, high-hierarchy nodes where the network becomes destabilized. We assume that similar critical points exist throughout various hubs. This conceptual framework, which we propose as the paradox of non-locality in cognitive mapping, suggests that the critical region does not inherently contain the function. Instead, it represents a hierarchically significant point whose perturbation is sufficient to induce behavioral deficits. Recognizing this would enable the preservation of higher-order networks during neurosurgical interventions.

When we apply DES to identify critical points while the patient performs cognitive tasks, a second or third-level neural disruption (Duffau, 2020) is generated in these concrete points, leading to a paradox: in that discrete area, a network—such as the default mode network during social cognition tasks—is distorted. However, we cannot directly conclude that point as “containing” the higher-order cognitive function under study. Instead, it should be considered a critical point where the network dynamics are interrupted, generating what we will term in this study the paradox of the non-locality of cognitive mapping (Figure 1A).

Understanding this conceptual paradox is crucial because (1) these critical points exhibit significant inter-individual and even intra-subject variability during mapping and remapping situations (Ng et al., 2023; Coletta et al., 2024); (2) DES during awake cognitive mapping is the gold standard for preserving higher-order networks by identifying those critical points (Ramírez-Ferrer et al., 2024); (3) the uncertainty obtained by reconstructing higher-order networks through resting-state fMRI, since we can reliably map the main hubs of each network but not these critical points that enable us to create a safe cortical map before performing the tumor resection (Figure 1B). Therefore, those critical points need to be identified *in vivo* during surgery, as DES is the most assertive way to preserve higher-order networks at the individual level (Coletta et al., 2024).

What represents those critical points from a network perspective? From the complex dynamic systems theory (Figure 1B), upon which we conceptually propose the paradox of the non-locality of cognitive mapping, two characteristics could explain these phenomena: (1) Hierarchy: In any complex system, not all points of influence carry the same weight in influencing other parts of the system (Briggs and Peat, 1989; Bassett and Gazzaniga, 2011), existing, therefore, certain points where the leverage effect on the rest of the system is greater (Boccaletti et al., 2006); and (2) the non-linear interactions (Figure 1A) among the different elements of the networks could explain how the electrical perturbation of these higher-hierarchy points affects other regions that, although distant, are functionally connected (butterfly effect) (Palmer et al., 2014). However, it is important to note that future studies are needed to analyze the electrical perturbations induced by DES and correlate them with the different kinds of behavioral impairments. This will help verify whether the framework we propose from the physics of dynamic systems can aid in deciphering the neural underpinnings of the higher-order cognitive networks that govern our behavior. It is currently challenging to electrically determine whether a low-frequency bipolar stimulus can disrupt the short-lived metastability and multistability that subserve higher-order cognitive functions (Mandonnet, 2021).

## The crucial role of white-matter tracts in large-scale network dynamics: connectome-stop points

Due to the localizationist trend that has guided clinical practice in recent decades, brain function has often been understood in terms of isolated and static cortical modules, overshadowing the importance of deep white matter in higher-order cognitive functions (Sarubbo et al., 2015; Filley and Fields, 2016). Due to novel imaging techniques such as diffusion tensor imaging (DTI), neuroanatomical studies (Martino et al., 2010; Mandonnet et al., 2017), and computational approaches (Vohryzek et al., 2022), we understood that white-matter tracts enable the emergence of higher-order cognitive functions by mediating the way the cortex is connected (Duffau, 2015; Filley and Fields, 2016). These findings have been supported *in vivo* by DES procedures, which overcome some of the limitations intrinsic to the other methods: (i) some DTI algorithms generate a high percentage of false streamlines, potentially leading to white-matter injuries (Christidi et al., 2016); (ii) the brain shift reported in some series ranges from millimeters to centimeters (Duffau, 2023); (iii) the inability to investigate tract function using the BOLD signal (Menon et al., 1999). All these factors combined make DES the only tool capable of providing *in vivo* information by inducing a transient dyssynchronization within the mosaic of cortical areas involved in one or multiple neural networks (Duffau, 2023). This brief disruption, lasting only a few seconds, elicits behavioral disturbances that the neuropsychologist can identify. This is critically important, as lesions to white matter tracts—unlike cortical lesions—have a limited potential for recovery due to low neuroplasticity, as described in white matter atlases (Herbet and Duffau, 2020). Consequently, preserving the integrity of these tracts during surgery is crucial for maintaining normal cognitive function. In this line, the surgical procedure is considered complete once these points are reached, recently proposed as “connectome-stop points” (Martín-Fernández et al., 2024), the surgical procedure is considered complete.

## Awake cognitive mapping in three steps from a dynamic and connectomic-centric perspective

Considering this paradigm shift, we propose understanding the brain as a complex dynamic system where: (1) Higher-order cognitive functions are emergent properties arising from the interaction of several large-scale networks (Thiebaut de Schotten and Forkel, 2022), for which it is crucial. (2) The preservation of deep white-matter tracts that enable synchronization across networks (Filley and Fields, 2016), to the extent that even small cuts in white matter can lead to disconnection syndromes (Herbet and Mandonnet, 2023), means we need to move from a modular-based perspective of awake surgery to a dynamic, individualized, and systems-level-based approach. To this end, an awake multimodal cognitive mapping in three phases with *in vivo* neuropsychological monitoring of higher-order cognitive functions—from social cognition to executive functions—from a meta-networking perspective (Duffau et al., 2022; Martín-Fernández et al., 2024) would allow us to achieve an optimal once-functional balance dynamically and assertively.

### Step 1: mapping critical regions of neural networks

In the first phase, we performed an electrical mapping of the critical regions of those networks around the tumor at risk of damage. Given the non-locality paradox of higher-order functions driven by constant across-network dynamics, it is necessary to identify the exact and discrete critical points within those networks by eliciting a behavioral impairment during the task. To find these critical points at the cortical surface, as mentioned above, DES is the gold standard. The mapping is performed following the widely known positive stimulation technique (Szelényi et al., 2010), in which the milliampere threshold is established upon generating a speech arrest in the ventral premotor cortex (VPMc). This approach minimizes the risk of electrically induced seizures while allowing a temporary distortion of neural networks to identify critical regions as the patient engages in various language, cognitive, and motor tasks.

### Step 2: multitask monitoring of cognitive functions during resection

A custom non-serial multitasking approach with a 4-s time constraint, designed around tumor-affected circuits, provides real-time information regarding intra-network and cross-network dynamics (Martín-Fernández et al., 2024). This enhances the sensitivity of behavioral monitoring, in accordance with the three-level neural disruption model (Duffau, 2020), by increasing cognitive demand throughout the resection and, therefore, optimizing the possibility of returning to work (Duffau et al., 2022). During this phase, time becomes the main limitation: after 1 h and a half, fatigue appears in the patient. The proposed constant multitasking approach would reveal or unmask the neural organization of the nervous system in terms of meta-networks and transient equilibrium states generated by specialized neural systems (Herbet and Duffau, 2020). This provides information on possible desynchronization between systems, allowing the neurosurgeon to adjust planning, proceed, or halt the surgery. In this way, we further optimize the once-functional balance achieved with awake surgery, which is necessary for the patient to return to a normal life.

### Step 3: identification and preservation of connectome-stop points

Even small injuries to the white-matter tracts during tumor resection could result in the disconnection of various brain areas, leading to the cessation of activity in one or several neural networks—for example, what might result from resecting the uncinate fasciculus and its contribution to the salience network or resecting the SLF-II and its implication in hemineglect provoked by attentional networks damage (Duffau, 2015; Herbet and Duffau, 2020)—. For all these reasons, we propose that this third phase—looking for the connectome-stop points—is necessary to guide the functional limits of our resection, thus addressing the key question of modern neuro-oncology: where to stop the resection given the fact that gliomas will constantly be growing, that is to say, there are no tumor limits? To achieve this, we must understand the behavioral impairment that DES will produce on each tract to have a better preoperative planification between neurosurgeons and neuropsychologists (Herbet and Moritz-Gasser, 2019; Martín-Fernández et al., 2024).

### Illustrative case: right inferior parietal lobe glioma

**Figure 2:**
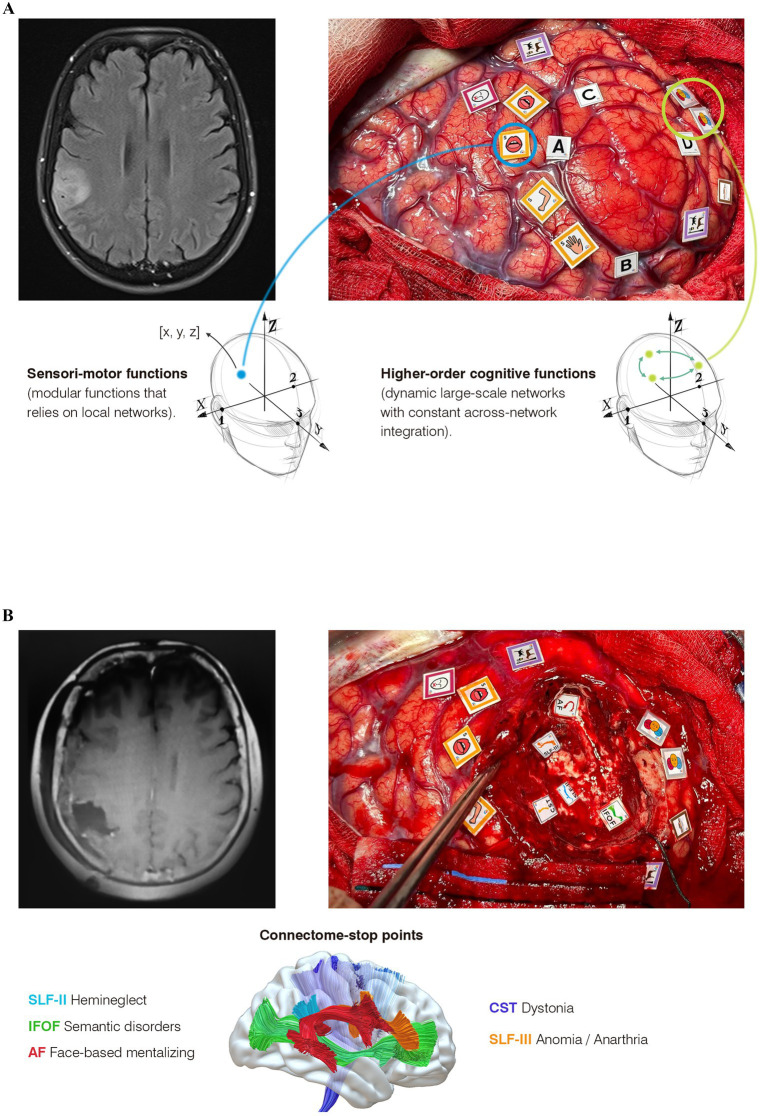
Multimodal cognitive mapping in three steps from a connectomic-centric perspective. **(A)** Images of awake cortical and subcortical cognitive mapping for a right parietal glioma. On the left, the postoperative MRI demonstrates complete resection with preservation of deep white matter. On the right, subcortical mapping shows the “connectome-stop” points, identifying each tract according to the behavioral impairments elicited by DES. Below, each observed behavioral impairment is explained in relation to its corresponding tract. **(B)** On the left, the preoperative MRI is suggestive of a glioma. On the right, cortical mapping reveals critical regions for sensorimotor functions (yellow tags), language (fuchsia tags for speech arrest, violet tags for unimodal semantics), and higher-order networks (three-face tags for emotion recognition and brown tags for hemineglect). Below, the differences in the neural underpinnings between mapping modular functions (such as left hemisensory regions in this case) and higher-order functions are highlighted: While modular functions (e.g., left hemisensory processing) imply that each critical region inherently controls sensitivity in these areas, allowing for the assignment of x, y, z coordinates, higher-order cognitive functions—such as emotion recognition—do not necessarily reside in these critical points. Rather, these points (also with their own coordinates) represent hypothesized network-critical nodes where electrical stimulation induces a transient disruption in network dynamics. This leads to a conceptual paradox for neurosurgeons, as the bipolar stimulator targets an apparently isolated, discrete point less than one centimeter in size, illustrating the need to distinguish between modular and cognitive mapping.

In this study, we present the case of a 66-year-old right-handed woman married without previous medical history who dedicates her life to her family and her work as a lawyer. After a first left faciobrachial motor seizure, an MRI was performed, showing a hyperintense signal on the FLAIR sequence in the right inferior parietal lobe without contrast enhancement, suggestive of a lower-grade glioma (Figure 2A). At that time, it was agreed with the patient to perform an awake surgery with multimodal cognitive mapping to achieve an optimal once-functional balance, considering the patient’s primary desire to resume her professional activity. The preoperative neuropsychological assessment revealed some impairments in social cognition: mild–moderate impairment in low-level mentalizing (Reading the Mind in the Eyes Test, or RME) and moderate in high-level mentalizing (assessed using the Movie for the Assessment of Social Cognition). No deficits were found in the remaining higher-order cognitive functions or sensorimotor functions (see Supplementary Table 1).

A right fronto-temporo-parietal craniotomy was performed. After opening the dura, the anatomical boundaries of the tumor were delineated using intraoperative ultrasound imaging (Figure 2A tags A, B, C). Cortical mapping began with counting numbers and flexion extension of the left upper limb. Stimulation of the VPMc elicited a speech arrest at 2 mA (Figure 2, fuchsia tag). Slightly posteriorly, at the level of the postcentral gyrus, DES-induced dysesthesias of the left hemiface and mouth correspond to the primary sensory area, with the arm area found dorsally (Figure 2A, yellow tags). After that, we commenced the cognitive mapping to find critical regions involved in higher-order functions such as spatial cognition, semantic cognition, and low-level mentalizing. Along the supramarginal gyrus, a critical region for unimodal semantics was identified using the Pyramids and Palm Trees (PPT) test (Figure 2A, violet tag). In the posterior part of the angular gyrus, DES-induced reproducible transient deficits in low-level mentalizing during the RME test in two cortical regions (three face tags): a reproducible distortion in the ability to recognize the emotion displayed on faces was observed in both sites. Slightly superiorly, a critical region for hemineglect was detected, evoking consistent deviations of 15 mm to the right during the Line Bisection Task (LBT). Every critical region was triple-checked in a non-consecutive manner to avoid stimulating the same area consecutively.

At this point, it seems crucial to highlight that although awake mapping has demonstrated its specificity and sensibility in identifying critical regions (Coletta et al., 2024), it is evident that cognitive mapping is constrained by the focal nature of stimulation sites and the inherent complexity of assessing higher-order functions intraoperatively, as many higher-order functions share overlapping cognitive subprocesses, which can sometimes make it challenging to associate critical points with specific cognitive functions (Königs et al., 2021). Consequently, a systems-based perspective of human brain function is essential to accurately utilize data from awake mapping to advance our understanding of higher-order processes. For this purpose, we proposed a perspective of a conceptual paradox of non-locality of higher-order functions striving to establish a bridge between computational neuroscience and awake mapping. This approach draws upon the principles of complex dynamic systems theory, a framework that has not been previously proposed from a neurosurgical perspective.

Following cortical mapping, a corticectomy was performed guided by the functional map while the patient engaged in tailored constant multitasking. This included left upper limb flexion and extension alternating between (1) LBT and Line Cancellation Test (LCT), (2) PPT test, and (3) RME plus self-evaluation (SCI) (Ng et al., 2021). The patient completed multitasking throughout the resection until we approached the deep white matter, where the connectome “stop-points” were identified (Figure 2B): (1) Posterior margin of the surgical cavity: the inferior fronto-occipital fasciculus (IFOF) was identified by inducing reproducible errors in unimodal semantics (PPT test) associated with loss of self-evaluation (SCI) during DES; (2) anteroinferior region: the arcuate fasciculus was identified through reproducible difficulties in face-based mentalizing (RME); (3) Slightly superior and superficially, the SLF-III was identified, eliciting both anarthria and anomia; and (4) deeper and posteriorly: three reproducible deviations of 25 mm to the right during LBT were provoked during DES probably related to SLF-II transient disconnection.

After experiencing a brief episode of transient allocentric left hemineglect (see Supplementary Figure 1), the patient was discharged with outpatient rehabilitation. The postoperative MRI showed a supratotal resection (Figure 2B). She resumed a normal social-familiar and professional life 6 weeks post-surgery. Detailed preoperative and postoperative neuropsychological assessments: day 0, 1 month, and 3 months post-surgery is provided in Supplementary Table 1.

## Limitations and perspectives

The presented paradox would be a useful conceptual tool to keep on disentangling neural and behavioral underpinnings of awake cognitive mapping and, as a result, a key to obtaining a better understanding of the relationship between complex network dynamics across spacetime and the emergence of higher-order functions. However, it should be noted that DES is an invasive method that can only be studied in patients with intracranial pathologies such as gliomas due to ethical reasons. Despite the well-known neural reorganization due to neuroplasticity and the limitation that this could suppose in drawing conclusions, previous large databases in patients with brain tumors have been shedding some light on connectomics (Sarubbo et al., 2015; Herbet and Duffau, 2020). Performing *in silico* experiments with those databases using novel computational approaches is a promising way to study the role of these critical points inside the different networks. However, the creation of reliable databases and a standardization of all the processes is mandatory. Current imaging and non-invasive mapping technologies still face significant temporal and spatial limitations, limiting their ability to capture the full complexity and temporal dynamics of brain networks. Lastly, the variability in individual brain connectomes adds another layer of complexity, making generalized conclusions difficult and emphasizing the need for personalized approaches. To address these challenges, new fMRI techniques under development aim to minimize spatiotemporal limitations by capturing the time-varying aspects of network dynamics, such as the dynamic conditional correlation approach or the sliding window correlation approach (Lindquist et al., 2014; Zalesky and Breakspear, 2015).

## Conclusion

A paradigm shift from a modular-based vision of brain functioning to a dynamic, connectomic-centric perspective is essential to enhance our understanding of higher-order functions, given its implication for neurosurgery to preserve the quality of life. The application of complex dynamic system theory would help us to fill the gap between computational neurosciences and neurosurgical practices, facilitating awake cognitive mapping that transcends glioma topography. The proposed three-phase cognitive mapping includes (1) identifying critical points from local to large-scale networks, (2) implementing dynamic non-serial multitasking during resection, and (3) identifying connectome-stop points. This approach appears to be the most effective method to dynamically and flexibly monitor brain functions within a complex systems-based framework.

## Data Availability

The original contributions presented in the study are included in the article/Supplementary material, further inquiries can be directed to the corresponding author.
