# Supplementary material for: Where are higher-order cognitive functions? The paradox of non-locality in awake cognitive mapping using a complex dynamic system framework
Source: Front Psychol. 2025 Mar 3;16:1542505. doi: 10.3389/fpsyg.2025.1542505 (PMC11922077; doi:10.3389/fpsyg.2025.1542505)
Supplement: Supplementary file 1 [file Presentation_1.pptx]

## Slide 1
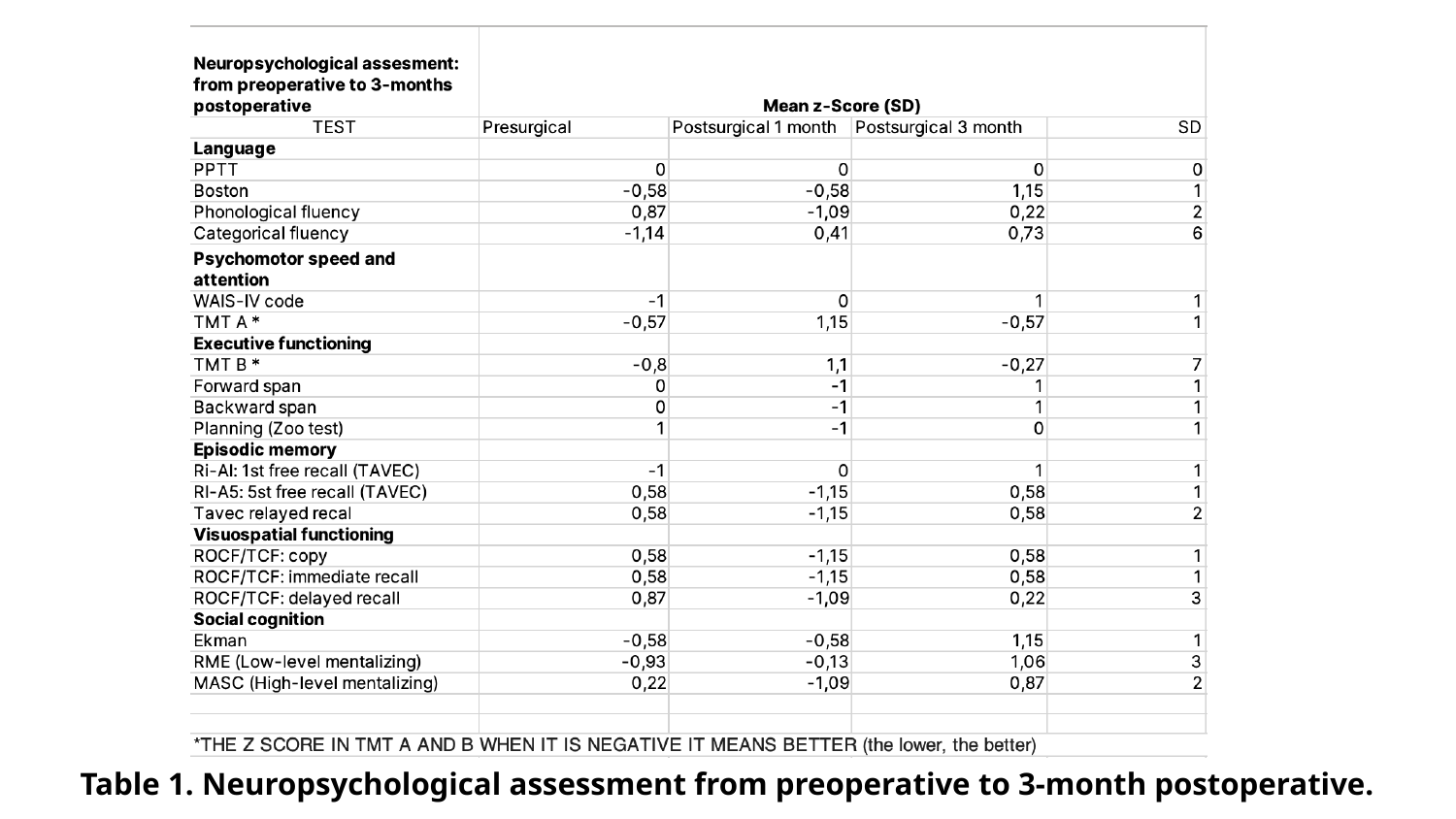

Table 1. Neuropsychological assessment from preoperative to 3-month postoperative.

## Slide 2
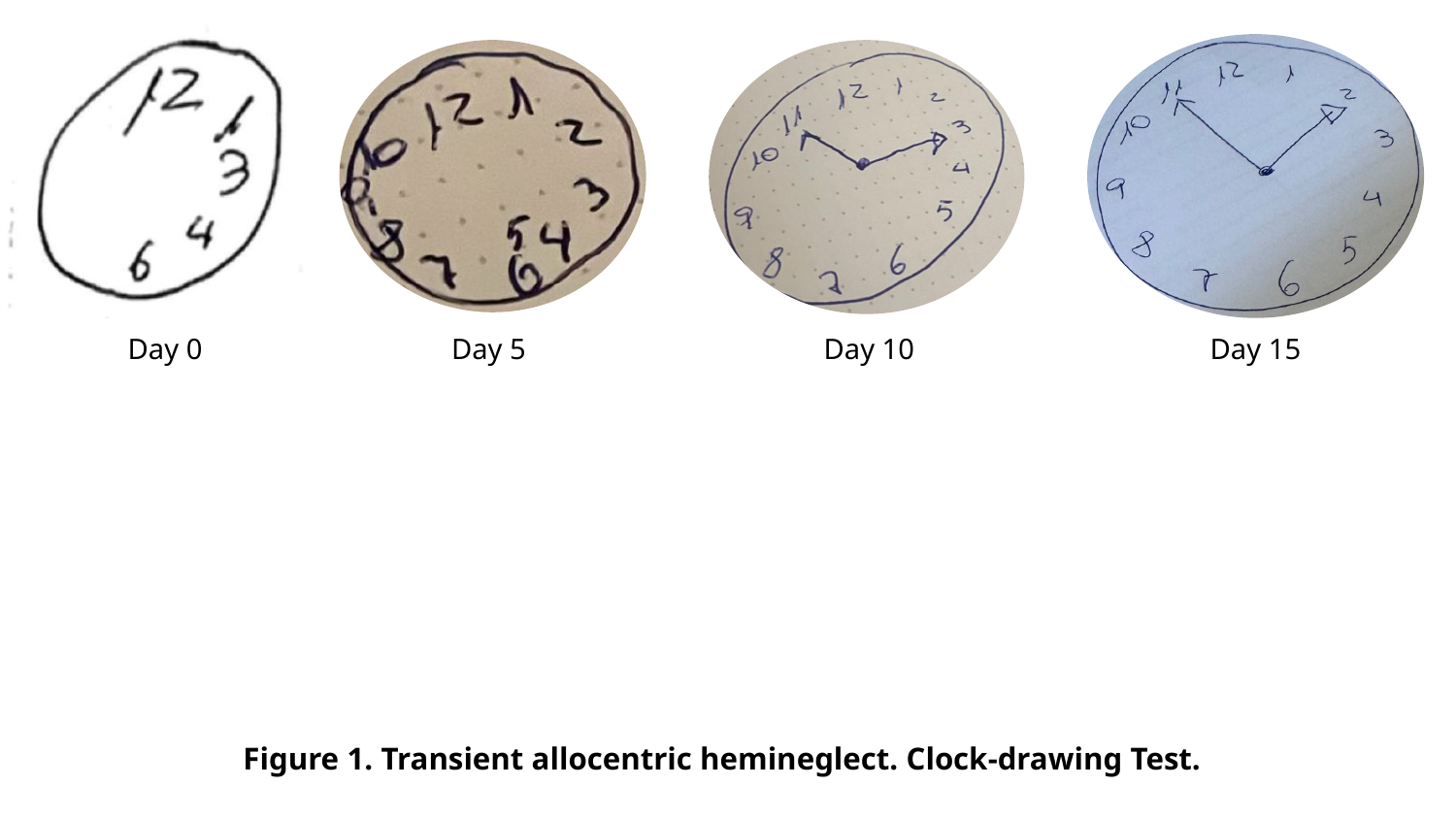

Day 10
Day 5
Day 0
Day 15
Figure 1. Transient allocentric hemineglect. Clock-drawing Test.

## Slide 3
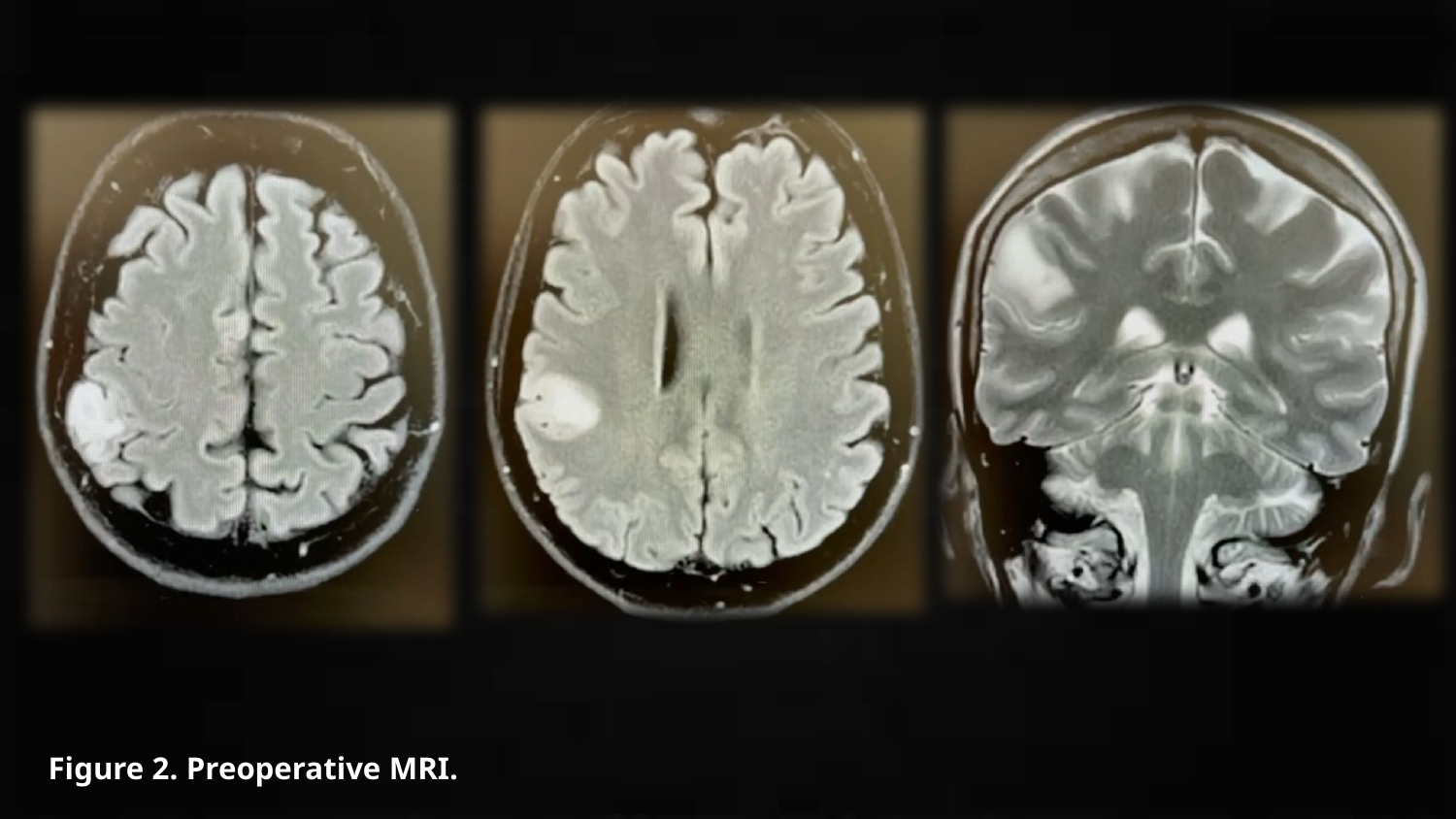

Figure 2. Preoperative MRI.

## Slide 4
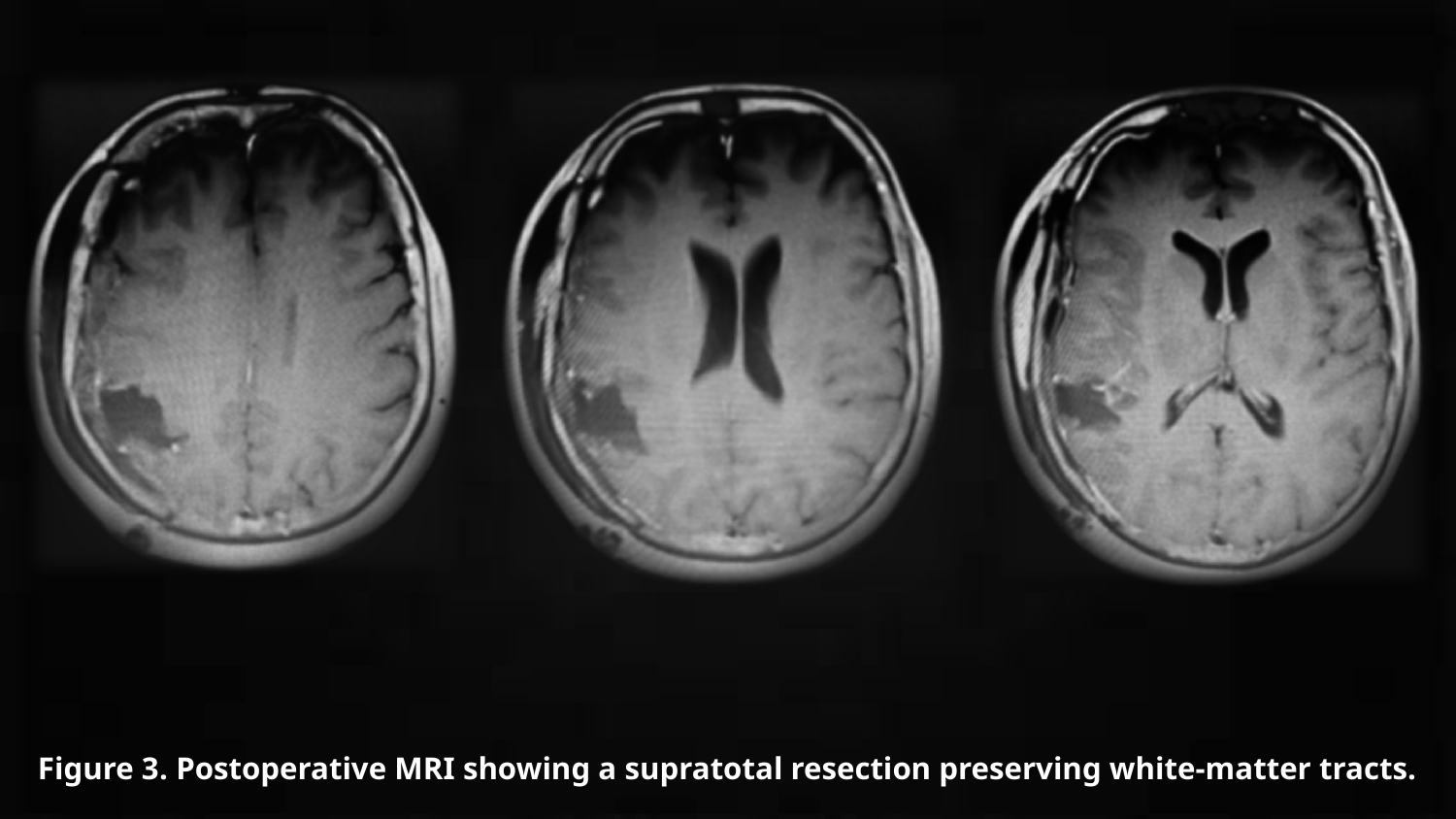

Figure 3. Postoperative MRI showing a supratotal resection preserving white-matter tracts.

## Slide 5
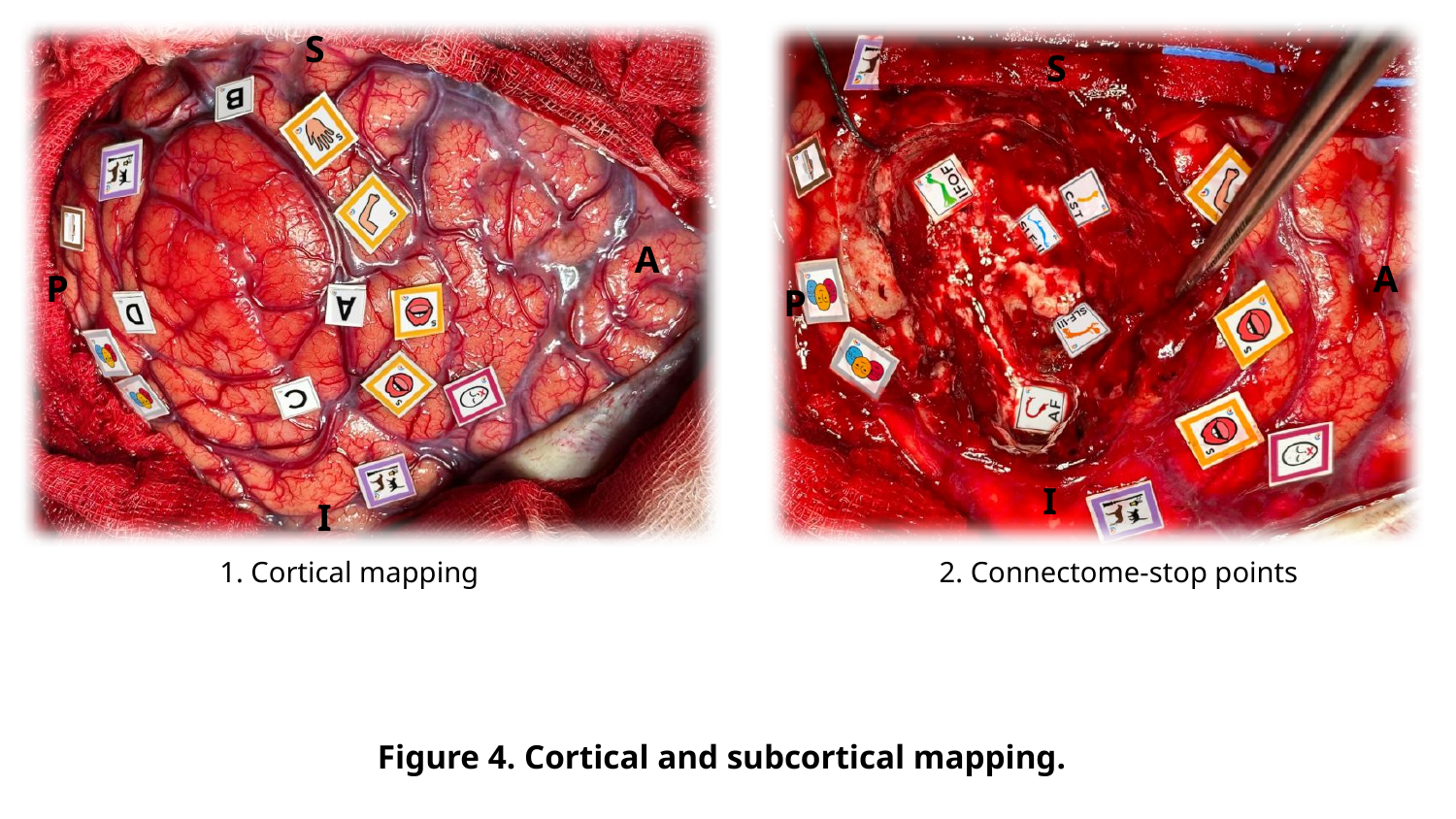

S
S
A
A
P
P
I
I
1. Cortical mapping
2. Connectome-stop points
Figure 4. Cortical and subcortical mapping.
